# Radiotherapy for painful shoulder syndrome: a retrospective evaluation

**DOI:** 10.1007/s00066-024-02302-x

**Published:** 2024-09-23

**Authors:** Ronny Leist, Oliver Micke, M. Heinrich Seegenschmiedt, Irenaeus A. Adamietz, Kashyar Fakhrian, Ralph Muecke

**Affiliations:** 1 Clinic for Orthopedics Wriezen, Wriezen, Germany; 2 Department of Radiotherapy and Radiation Oncology, Franziskus Hospital Bielefeld, Bielefeld, Germany; 3 Radiotherapy Duisburg, Duisburg, Germany; 4 Department of Radiotherapy and Radiation Oncology, Marien Hospital Herne, Ruhr, University Bochum, Bochum, Germany; 5 Radiotherapy Aschaffenburg, Aschaffenburg, Germany; 6 Radiotherapy RheinMainNahe, Bad Kreuznach, Mainz, Rüsselsheim, Germany

**Keywords:** Benign diseases, Low-dose radiotherapy, Linear accelerator, Orthovoltage device, Good treatment success

## Abstract

**Purpose:**

We evaluated the efficacy of low-dose radiotherapy for painful shoulder syndrome from an orthopedic perspective.

**Methods:**

Patients with painful shoulder syndrome were recruited for this retrospective clinical quality assessment from January 2011 to December 2017. Patients were treated with a linear accelerator or an orthovoltage device at individual doses of 0.5–1.0 Gy and total doses of 3.0–6.0 Gy. To assess response, we used the von Pannewitz score with five levels: “worsened,” “unaffected,” “improved,” “significantly improved,” and “symptom free.” “Good treatment success” was defined as “significantly improved” and “symptom free.” Within-group and between-group differences were statistically evaluated.

**Results:**

Of 236 recruited patients (150 women, 86 men; mean age 66.3 [range 31–96] years), 180 patients underwent radiotherapy with a linear accelerator and 56 with an orthovoltage device. Fractionation was 12 × 0.5 Gy in 120 patients, 6 × 0.5 Gy in 74, and 6 × 1 Gy in 42 patients. Treatments were completed in one series for 223 and in two series at least 6 weeks apart for 13 patients. Of the 236 patients, 163 patients (69.1%) agreed to be re-interviewed at a median of 10.5 (range 4–60) months after radiotherapy completion. Directly after radiotherapy, 30.9% (73 patients) had “good treatment success,” which had increased to 55.2% (90 patients) at follow-up.

**Conclusion:**

Protracted pain improvement with low-dose radiotherapy is possible in painful shoulder syndrome. Patients with refractory pain because of subacromial syndrome or shoulder osteoarthritis should also be evaluated for radiotherapy.

## Background

Painful shoulder syndrome is common, with a lifetime prevalence of up to 66.7% [1]. The frequency of chronic shoulder complaints and the socioeconomic aspects are reflected in health insurance company reports, such as one published in 2021 showing that in 2020, 15.7% of employees subject to social insurance contributions reported complaints due to shoulder syndrome. The diagnostic subgroup “shoulder lesions” according to the International Statistical Classification of Diseases and Health-Related Health Problems, tenth revision (ICD-10) [2], caused 20.18 days of incapacity for work per 100 insured person years, placing this diagnosis 13th among three-digit ICD-10 codes in 2021 and third among all diseases of the musculoskeletal system and connective tissue [3].

Shoulder pain syndrome is a broad term encompassing various conditions including subacromial syndrome in its multiple manifestations, acromioclavicular arthrosis, and enthesopathies, each characterized by distinct pathophysiological mechanisms and clinical presentations. Standard radiographs are fundamental to diagnosing shoulder pain syndrome [4]. They often reveal subchondral sclerosis of the major tuberosity, subacromial spurs, acromial shape anomalies, and acromioclavicular joint arthrosis. These radiographs are crucial for the differential diagnosis, identifying calcifying tendinitis, fractures, and neoplasms. Magnetic resonance imaging (MRI) is generally considered the preferred method for assessing the rotator cuff and adjacent structures [5].

Because of the high burden of related illness, numerous therapeutic measures and strategies have been developed and optimized for shoulder pain. Despite these predominantly operative and conservative treatment options, many patients live with an unsatisfactory outcome or cannot be treated surgically [6]. Another option is low-dose radiotherapy, which has become established for the treatment of osteoarthritis and enthesiopathies, including painful shoulder syndrome [7–16].

Our objective was to broaden the evidence base concerning the use of radiotherapy for painful shoulder syndrome, specifically examining the analgesic effects of low-dose irradiation from an orthopedic viewpoint.

## Methods

### Patients and treatment

Patients with painful shoulder syndrome were recruited for this retrospective clinical quality assessment.

The data were collected during 2011 to 2013 at Klinikum Lippe in Lemgo and in 2017 at RheinMainNahe Radiotherapy using linear accelerators at both sites and the orthovoltage device at Klinikum Lippe, with different fractionation schemes. All patients provided informed consent regarding radiotherapy and participation in this clinical quality assessment prior to enrollment.

In our study, 197 of 236 patients (83.5%) were referred by orthopedic surgeons after undergoing a clinical examination that included X‑ray for all patients and MRI for 34 patients (14.4%). Thirty-nine patients (16.5%) were referred by family physicians and had a prolonged history of treatments extending over more than 6 months, involving various interventions suggested and performed by both orthopedic surgeons and family physicians. All 39 patients underwent X‑ray imaging, and 28/39 (71.7%) also received MRI. Overall, the duration of pain before initiating radiotherapy varied, with 71 patients experiencing pain for more than 3 months but less than 6 months, and 165 patients for longer than 6 months. Radiation therapy was the primary treatment choice in only 20 cases; the remaining 216 patients had received multiple pretreatments, including local injections, oral cortisone, nonsteroidal anti-inflammatory drugs (NSAIDs), shockwave therapy, and physiotherapy.

Patients were treated with either the linear accelerator or the orthovoltage device. The single doses amounted to 0.5 to 1.0 Gy and the total doses to 3.0 to 6.0 Gy.

Prior to radiation therapy on the linear accelerator, 120 patients underwent simulator-based and 60 patients three-dimensional computer tomographic (CT)-based treatment planning. In the 56 patients who were treated with the orthovoltage device, radiation therapy was carried out after clinical adjustment on the device with the 8 × 8 cm and 10 × 10 cm tube. After simulator-based and CT planning, the field sizes were determined individually depending on the size of the patients. In each case, the lungs and, in women, also the breasts were shielded.

To assess the response to treatment directly after radiotherapy and at follow-up, we used the von Pannewitz score consisting of five categorical levels: “worsened,” “unaffected,” “improved,” “significantly improved,” and “symptom free.” “Good treatment success” according to this score was defined as “significantly improved” and “symptom free.”

The assessment of analgesic efficacy was carried out by a telephone survey. All results were recorded in an Excel (Microsoft, Redmond, WA, USA) spreadsheet and then transferred to SPSS (IBM Corp., Armonk, NY, USA) for evaluation after completion of the survey.

### Statistical analysis

All data were stored and analyzed using the SPSS statistical package v. 23.0. Descriptive statistics were computed for continuous and categorical variables, including median and interquartile ranges of ordinal variables, mean and standard deviations of continuous variables, and frequencies and relative frequencies of categorical factors. The Wilcoxon signed-rank test was used to test for differences in continuous and categorical variables within the groups. To test for between-group differences, we used the Mann–Whitney U test for continuous variables and Fisher’s exact test for categorical variables, as appropriate. All *-*values were two-sided statistical tests, and *P* < 0.05 was considered significant.

## Results

### Patients

A total of 236 evaluable patients (150 women, 86 men) with a mean age of 66.3 years (range 31–96 years) were recruited. Radiotherapy was carried out using a linear accelerator in 180 patients and an orthovoltage device in 56 patients. A total of 120 patients were treated with a fractionation scheme of 12 × 0.5 Gy, 74 patients with 6 × 0.5 Gy, and 42 patients with 6 × 1 Gy. In 223 patients radiation treatments were completed in one series, and in 13 patients they were completed in two series at least 6 weeks apart. No side effects were observed. Of the 236 patients, 163 (69.1%) agreed to be re-interviewed at a median of 10.5 months (range 4–60 months) after the end of radiotherapy.

### Treatment success: within-group comparisons

Directly after radiotherapy, 30.9% (73/236) experienced good treatment success, which had increased to 55.2% (90/163 patients) at the time of follow-up care (*P* < 0.001; Table 1).

**Table 1:** Von Pannewitz score survey results directly after therapy and at follow-up in the overall patient cohort

Timing (*n*)	Symptom free, *n* (%)	Significantly improved, *n* (%)	Improved, *n* (%)	Unaffected, *n* (%)	Worsened, *n* (%)
End of radiotherapy (*n* = 236)	8 (3.4)	65 (27.5)	96 (40.7)	57 (24.2)	10 (4.2)
Follow-up (*n* = 163)	29 (17.8)	61 (37.4)	33 (20.2)	35 (21.5)	5 (3.1)

Table 2 shows the von Pannewitz score results by treatment device. Among those receiving therapy with a linear accelerator, 27.2% (49/180 patients) experienced good treatment success at the end of radiotherapy, compared with 52.6% (71/135 patients) at follow-up. The increase between these two timepoints was highly significant (93.4%, *P* < 0.001). For patients receiving treatment with an orthovoltage device, 42.9% (24/56 patients) experienced good treatment success directly after radiotherapy ended, which increased to 67.9% (19/28 patients) at follow-up. The increase between these timepoints was not significant (58.3%; *P* = 0.157).

**Table 2:** Von Pannewitz score survey results directly after therapy and at follow-up, by device

Device and timepoint (*n*)	Symptom free, *n* (%)	Significantly improved, *n* (%)	Improved, *n* (%)	Unaffected, *n* (%)	Worsened, *n* (%)
Linear accelerator, end of radiotherapy (*n* = 180)	5 (2.8)	44 (24.4)	75 (41.7)	48 (26.7)	8 (4.4)
Linear accelerator, follow-up (*n* = 135)	21 (15.6)	50 (37.0)	31 (23.0)	28 (20.7)	5 (3.7)
Orthovoltage device, end of radiotherapy (*n* = 56)	3 (5.4)	21 (37.5)	21 (37.5)	9 (16.1)	2 (3.5)
Orthovoltage device, follow-up (*n* = 28)	8 (28.6)	11 (39.3)	2 (7.1)	7 (25.0)	0 (0.0)

Table 3 shows von Pannewitz score results by fractionation scheme. With the 12 × 0.5 Gy fractionation scheme, 31.7% (38/120 patients) had good treatment success at the end of radiotherapy, compared with 57.2% (40/70 patients) at follow-up (80.1% increase; *P* < 0.001). Among patients receiving 6 × 1.0 Gy, 35.8% (15/42 patients) had good treatment success directly after the therapy, compared with 73.7% (14/19 patients) at the follow-up, for an increase of 106.4% (*P* = 0.102). For those receiving 6 × 0.5 Gy, 27.1% (20/74 patients) had good treatment success just after therapy and 48.7% (36/74 patients) did so at follow-up. This increase also was highly significant (80.0%; *P* < 0.001).

**Table 3:** Von Pannewitz score survey results directly after therapy and at follow-up for each fractionation scheme

Scheme and timepoint (*n*)	Symptom free, *n* (%)	Significantly improved, *n* (%)	Improved, *n* (%)	Unaffected, *n* (%)	Worsened, *n* (%)
12 × 0.5 Gy, end of radiotherapy (*n* = 120)	5 (4.2)	33 (27.5)	45 (37.5)	33 (27.5)	4 (3.3)
12 × 0.5 Gy, follow-up (*n* = 70)	21 (30.0)	19 (27.2)	12 (17.1)	18 (25.7)	0 (0.0)
6 × 1 Gy, end of radiotherapy (*n* = 42)	2 (4.8)	13 (31.0)	19 (45.1)	7 (16.7)	1 (2.4)
6 × 1 Gy, follow-up (*n* = 19)	5 (26.3)	9 (47.4)	2 (10.5)	3 (15.8)	0 (0.0)
6 × 0.5 Gy, end of radiotherapy (*n* = 74)	1 (1.4)	19 (25.7)	32 (43.2)	17 (23.0)	5 (6.7)
6 × 0.5 Gy, follow-up (*n* = 74)	3 (4.1)	33 (44.6)	19 (25.7)	14 (18.8)	5 (6.8)

### Treatment success: between-group comparisons

Among patients receiving therapy with the orthovoltage device, 42.9% experienced treatment success directly after therapy, compared with 27.7% undergoing treatment with the linear accelerator (*P* = 0.033). The two groups did not differ at follow-up, with 67.9% experiencing treatment success with the orthovoltage device compared to 52.6% for the linear accelerator (*P* = 0.141).

For the different fractionation regimens, the percentages of patients experiencing treatment success directly after therapy did not vary among the groups (32.5% for 12 × 0.5 Gy, 35.7% for 6 × 1.0 Gy, 26.8% for 6 × 0.5 Gy; *P* = 0.353). The groups also did not differ significantly at follow-up (57.1% for 12 × 0.5 Gy, 73.7% for 6 × 1.0 Gy, 48.6% for 6 × 0.5 Gy; *P* = 0.136).

### Sex-based differences

Women and men did not differ in treatment success rates either directly after therapy (women 29.7% vs. men 34.1%; *P* = 0.488) or at follow-up (women 54.4% vs. men 56.7%; *P* = 0.777).

## Discussion

The results of our retrospective clinical quality assessment confirm findings from recently published retrospective and prospective randomized studies showing a good analgesic effect of low-dose radiotherapy in patients with painful shoulder syndrome.

According to Ott et al., who reviewed 25 relevant studies published from 1943 to 2012 and covering 2928 (range 21–546) patients, the overall response rate was 82% (59%–96%): 40% (6%–79%) experienced complete symptom remission and 42% (15%–83%) had a partial remission [11, 13]. The wide ranges in these results reflect in part the different periods of data collection and in part the different treatment regimens and measurement instruments used for the evaluation.

The response rates among our patients of 71.6% directly after radiotherapy and 75.5% at the time of follow-up care are in line with earlier results (Table 1). The 3.4% experiencing complete remission directly after radiotherapy, however, is proportionally lower than some other findings, but the 17.8% having remission at follow-up appears to be more in line with the results of other studies (Table 1). The range of complete remissions is quite wide, indicating the necessarily subjective basis of each patient’s assessment of the response to therapy and the resulting classification.

In this study, we found no significant difference among the three low-dose fractionation regimens. These results are consistent with and confirm those of the Erlangen dose optimization study and the recommendations of the German Society for Radiation Oncology (DEGRO) [11–13].

There is a lack of consensus data regarding the advantages of different treatment devices in producing improved short- and long-term outcomes [16, 17]. Given the restricted patient sample size in our analysis, it is not feasible to conclusively favor one therapeutic device over another. Our study found that there was a higher number of individuals who experienced successful treatment with the orthovoltage device compared to linear accelerators immediately after radiation therapy. However, it is important to interpret these data with caution. The observed outcomes could potentially be attributed to use of the orthovoltage device at only a single location and involving a limited number of patients, in contrast to the deployment of linear accelerators at several sites. Large-scale comparative studies are necessary to definitively assess the influence of treatment equipment selection on the effectiveness of radiation therapy.

In contemporary orthopedic practice, a comprehensive understanding of various treatment modalities is crucial, particularly as the population ages and the incidence of degenerative shoulder conditions rises. Reviews in orthopedics suggest that treatment strategies for shoulder pain syndrome typically include NSAIDs, exercise therapies, steroid injections, and surgery for severe or refractory cases. However, many patients are not suitable candidates for surgical intervention due to a range of medical and personal factors. Notably, low-dose radiation therapy, as a conservative treatment option prior to considering surgery, is infrequently or not at all discussed in these reviews [18, 19]. Our research shows a positive response to low-dose radiation therapy in 55.2% of patients, highlighting its potential to significantly improve quality of life for patients in whom surgery is not feasible. Therefore, it is critical for orthopedic surgeons to consider low-dose radiation therapy as a viable treatment alternative. This approach not only broadens the scope of patient care but also promotes a multidisciplinary treatment strategy.

Concerns regarding the carcinogenic potential of radiation therapy for benign diseases occasionally deter its use. It is important to recognize that the bulk of data indicating a risk of cancer following radiation therapy for benign conditions are derived from outdated studies focusing on pediatric and young adult populations, who are no longer candidates for such treatments [20]. Current evidence suggests that the risk of cancer development after radiation therapy for benign diseases in middle-aged and older adults is exceedingly low. McKeown et al., in their seminal work investigating the carcinogenicity associated with radiation therapy for benign diseases, note in their conclusion that assessing the risk of radiation therapy for benign conditions presents numerous challenges, as the evidence base is limited and often derived from a disparate array of sources that are not directly comparable. They emphasize that the risk of radiation-induced cancer in older adults treated with radiation therapy for benign conditions, particularly those affecting peripheral tissues, is very small and diminishes further with age.

### Limitations

For some subgroups, the sample size was small; for example, only 28 patients could be recruited for follow-up treatment with the orthovoltage device and only 19 patients for follow-up treatment with 6 × 1.0 Gy. These small numbers may have affected the significance of increases in good treatment success.

One caveat of this study is that the exact diagnosis was not documented. Painful shoulder syndrome comprises several entities that differ in terms of pathogenesis and course, and the distribution of these diagnoses may not be the same between our patient population and the overall population with painful shoulder syndrome. In addition, radiotherapy may have variable effects depending on the specific shoulder condition. How these conditions differed in proportion between treatment devices and among fractionation regimens is not clear and could have been a factor in the significant advantage of the orthovoltage device.

Regarding the timing of treatment and previous and concomitant therapies in the overall course of the disease, this analysis can offer no precise conclusions. It is possible that some data were missing or incomplete from pretreatment physicians or that patient information was inaccurate, thus affecting the results and the comparability with other studies.

Furthermore, the response to radiotherapy in this study is based solely on pain perception and subjective report. Objective function scores, such as improvement in shoulder mobility, were not investigated. Finally, a placebo group with a similar composition was not included to exclude the effects of spontaneous changes or concomitant therapies [21–23].

## Conclusion

In line with previous literature, the present study shows that pain improvement in patients with painful shoulder syndrome is possible with low-dose radiotherapy, as is a protracted effect. Patients with refractory shoulder pain resulting from subacromial syndrome or osteoarthritis in the shoulder area should also be evaluated for radiotherapy.

## Data Availability

Please contact the corresponding author for data requests.
